# Ventricular Arrhythmia Catheter Ablation Following Anthracycline Exposure: A Single-Center Experience

**DOI:** 10.1016/j.jacadv.2025.101817

**Published:** 2025-06-09

**Authors:** Narut Prasitlumkum, Joerg Hermann, Nicholas Y. Tan

**Affiliations:** Mayo Clinic, Rochester, Minnesota, USA

Anthracyclines, including doxorubicin, daunorubicin, epirubicin, and idarubicin, are among the most effective chemotherapeutics utilized in particular in hematological malignancies, ovarian and breast cancers, and sarcoma. Cardiotoxicity has been one of the best known therapy-limiting side effects, usually presenting with a decline in left ventricular ejection fraction (LVEF) and heart failure. Arrhythmias can be observed among patients who undergo implantable cardioverter-defibrillator implantation for primary/secondary prevention, no difference in arrhythmia burden was seen when comparing anthracycline cardiomyopathy with either ischemic or nonischemic cardiomyopathy.^1^

The presence of ventricular arrhythmia (VA), including premature ventricular contraction (PVC) and ventricular tachycardia (VT), confers an increased risk of cardiovascular morbidity and mortality.^2^^,^^3^ The mechanisms linked to the negative impacts included PVC-induced cardiomyopathy and arrhythmic sudden cardiac death. For treatment modalities, catheter ablation has been commonly employed as a standalone or in combination with antiarrhythmic drugs for rhythm control.^2^ However, there are limited data regarding catheter ablation in patients with VA following anthracycline exposure, thereby prompting the pursuance of the following study.

This retrospective single-center study was approved by the Mayo Institutional Review Board. We screened patients >18 years seen at the Mayo Clinic Enterprise who received anthracycline-based therapy and underwent catheter ablation for VA from 01/2016 to 01/2024. A total of 8 patients were identified: 5 men and 3 women, with a median age of 69.5 years (IQR: 25%-75% Q1-Q3 45.5-73.5). The median time from first exposure to anthracycline to first report of VA was 36 months (Q1-Q3 18.5-123). Median LVEF was 45% with Q1-Q3 of 36% to 53.5%. Two patients were diagnosed with coronary artery disease requiring percutaneous coronary intervention to left anterior descending artery due to severe stenosis, but these were thought to not be contributing to cardiomyopathy or VA. Of the 8 patients, 3 patients underwent device implantation: 1 dual chamber pacemaker for complete heart block, 1 dual chamber implantable cardioverter-defibrillator for primary prevention, and 1 biventricular pacemaker with defibrillator for nonischemic cardiomyopathy and secondary prevention. Additionally, 3 patients had prior chest radiation. Cumulative anthracycline dosages were available for 2 patients (150 and 170 mg/m^2^, respectively).

**Figure 1:**
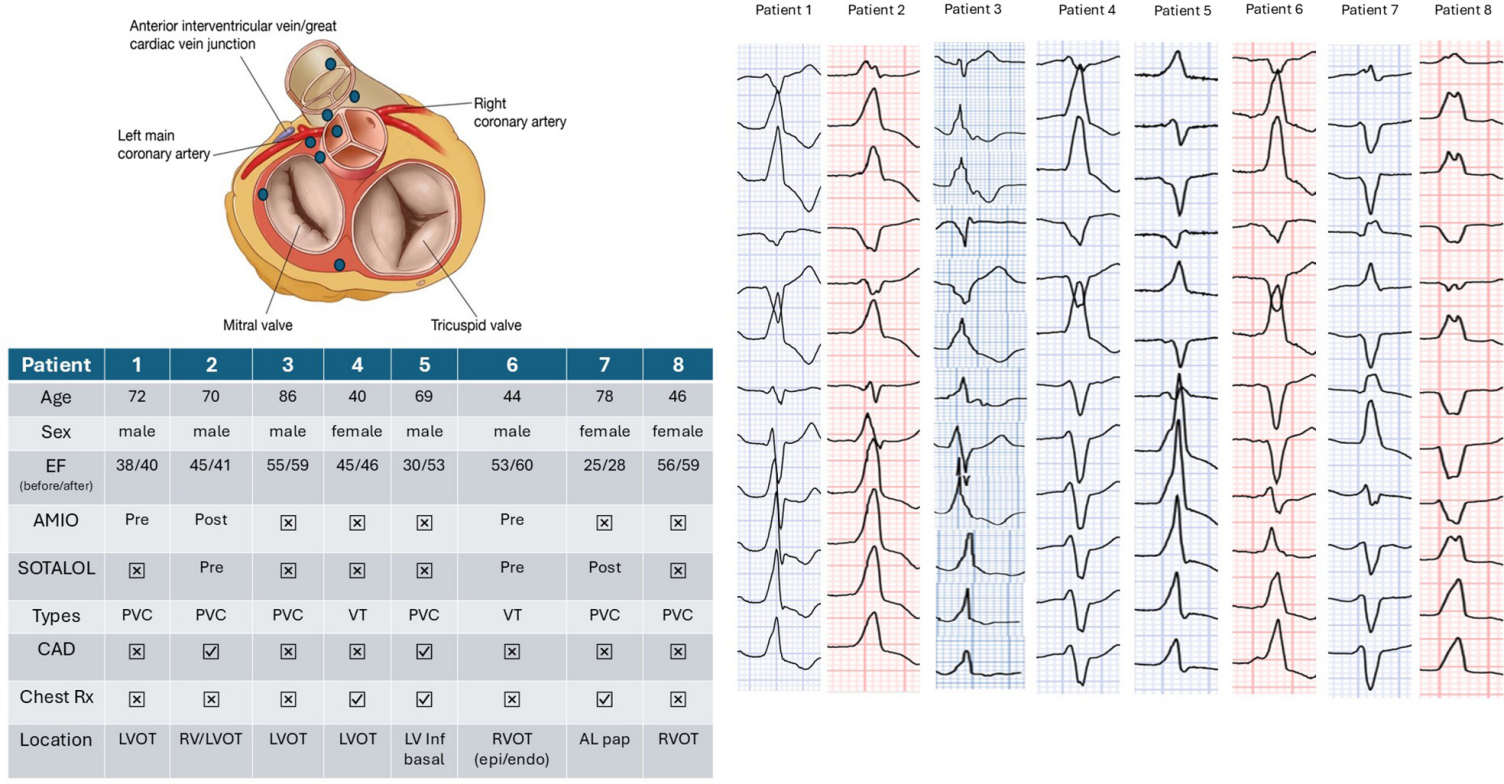
Demonstration of PVC Morphologies and Demographic Data From 8 Patients in our Registry Plum dots represented location of each PVC. AL = anterolateral; AMIO = amiodarone; CAD = coronary artery disease; EF = ejection fraction; LV = left ventricular; LVOT = left ventricular outflow tract; PVC = premature ventricular contraction; RVOT = right ventricular outflow tract; VT = ventricular tachycardia.

Four of the 8 patients were on antiarrhythmic therapy including sotalol and amiodarone prior to VA catheter ablation. As displayed in the Figure 1, each medication was used in 3 patients (2 in preablation and 1 in postablation phase). Those who received amiodarone had successful VA suppression but later discontinued antiarrhythmic therapy out of concerns for long-term side effects. Six of the 8 patients underwent catheter ablation for symptomatic PVC, whereas 2 were treated for sustained VT. Of the arrhythmias in this patient cohort, 6 were outflow tract in origin, 1 was basal left ventricular (LV) inferoseptum, and 1 was anterolateral papillary muscle. Seven of the 8 patients underwent only endocardial ablation, and 1 received combined epicardial/endocardial ablation. In 2 patients, abnormal substrate including low voltages and fractionated signals were noted in the regions of interest: 1 in the basal LV inferoseptum and 1 in the anteroseptal right ventricular outflow tract (both epicardial and endocardial). Pace mapping and activation mapping of VAs were utilized in all cases. Acute success was achieved in 6/8 (75%) of cases. Patients were followed for a median of 23.5 months (Q1-Q3 12.5-45.25). During follow-up, only one patient had substantial improvement in LVEF, from 30% to 53%, (Figure 1). Two patients required a redo procedure, 11 and 26 months, respectively, after the first unsuccessful ablation attempt. In one of these 2 patients, cardiac magnetic resonance imaging (MRI) identified an abnormal epicardial substrate, which was crucial in accurately defining the site of successful ablation. No complications were found in all patients during both the index and redo procedures. Two patients required long-term antiarrhythmic agents, one with sotalol and one with amiodarone.

To our knowledge, this is the first series on VA ablation in cancer patients who had undergone anthracycline therapy. Of the 8 patients identified, 5 had LVEF<50%. These were attributed to nonischemic cardiomyopathy in association with anthracycline exposure. All of these patients were long-term cancer survivors, presenting on average 3 years after anthracycline therapy for symptomatic VA requiring catheter ablation. Ventricular outflow tracts were the most common site for VAs which was similar to noncancer patients,^4^ followed by LV basal inferoseptum and anterolateral papillary muscle. The approaches to mapping and catheter ablation—activation/pace mapping and assessment of substrate abnormalities—were consistent with those used in other patient populations. Epicardial access might be required in addition to endocardial approach for complete VA suppression. Given the high acute success rates, catheter ablation appears to be a viable option for treating VAs in this setting.

As exemplified by one patient in our series, multimodal imaging might be useful to delineate the substrate associated with VAs in patients with anthracycline-associated cardiomyopathy. Cardiac MRI has been used to identify the prevalence and distribution of fibrosis (as determined by late gadolinium enhancement) following anthracycline use, but not without debate.^5^ Surprisingly, the patient in whom cardiac MRI did define the substrate did not have prior chest radiation exposure, while those with radiation exposure only had endocardial substrates. Of note, only 1 of the 5 patients with an LVEF <50% had a significant LVEF improvement. It is plausible that this patient had true PVC-induced cardiomyopathy while the other patients may have had irreversible myocardial damage.

This was a case series from a single tertiary referral center, and so selection bias cannot be ruled out. Additionally, the study was conducted in a single arm fashion, hence no comparison was performed. Larger studies and comparison analyses with noncancer patients are warranted to further characterize the safety, efficacy, and long-term outcome of cancer patients undergoing VA ablation. Finally, no dose-response relationship analysis could be performed due to limited information available regarding cumulative anthracycline dosages.
